# Sleep restriction increases reward sensitivity during sequential updating

**DOI:** 10.1093/sleep/zsaf354

**Published:** 2025-11-06

**Authors:** Jeryl Y L Lim, Daniel Bennett, Sean P A Drummond

**Affiliations:** School of Psychological Sciences, Monash University, Melbourne, Victoria, Australia; Turner Institute for Brain and Mental Health, Monash University, Melbourne, Victoria, Australia; School of Psychological Sciences, Monash University, Melbourne, Victoria, Australia; Melbourne School of Psychological Sciences, University of Melbourne, Melbourne, Victoria, Australia; School of Psychological Sciences, Monash University, Melbourne, Victoria, Australia; Turner Institute for Brain and Mental Health, Monash University, Melbourne, Victoria, Australia

**Keywords:** sleep restriction, sequential updating, decision making, reinforcement learning, Bayesian

## Abstract

Chronic sleep restriction is a common phenomenon in the real world, yet its impact on how individuals learn and adapt to dynamic patterns of reward in uncertain environments are not well understood. The present study examined how sleep restriction alters the cognitive mechanisms underlying sequential updating, defined as the process by which individuals revise beliefs over time in response to outcome feedback. Thirty-six healthy adults completed two conditions in counterbalanced order: well-rested (seven nights of 9 h time-in-bed) and sleep restriction (seven nights of 5 h time-in-bed). On the final day of each condition, participants performed a probabilistic reversal learning task, requiring flexible behavioral adjustments in response to changing reward contingencies. Computational models grounded in reinforcement learning and Bayesian frameworks were fitted to trial-level data using hierarchical Bayesian estimation. Our model comparison indicated the Asymmetric Rescorla–Wagner model best accounted for behavior, indicating feedback-driven updating with distinct learning rates for reward and nonreward outcomes on the probabilistic reversal learning task. Importantly, sleep restriction increased sensitivity to reward feedback and choice stochasticity, indicating greater reward-chasing behavior and less goal-directed decision making under sleep restriction. Trial accuracy did not differ between conditions. These findings diverge from previous evidence indicating reduced sensitivity toward negative outcomes following short-term sleep restriction, pointing to possible dose-dependent sleep restriction effects and those arising from differences in task requirements. Our findings may have practical implications for decision quality in real-world domains where performance depends on optimal feedback-based updating under uncertain decision environments.

**Statement of Significance**

While sleep loss is known to affect decision making, little is understood about how it impacts people’s ability to learn from experience over time. In many real-world situations, like financial trading and medical contexts, optimized decisions rely on updating beliefs and strategies based on incoming feedback. In this study, we show after one week of sleep restriction, individuals became more influenced by rewards and demonstrated increased choice randomness. This suggests they were more likely to chase immediate gains but had difficulty sustaining consistent decision strategies. These findings help explain how sleep restriction can undermine learning and flexible decision making when faced with uncertainty, and carry implications for performance and accountability in roles where repeated, high-stakes decisions are required.

## Graphical Abstract

**Graphical Abstract:**
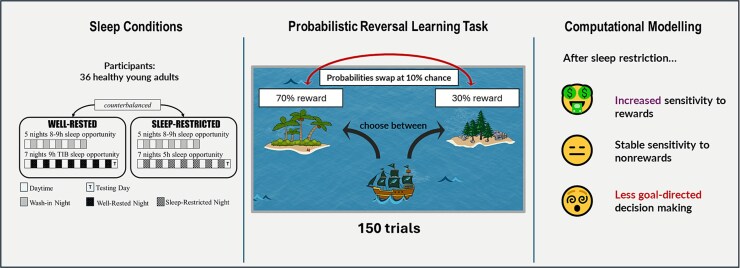

## Introduction

In today’s fast-paced world, chronic sleep restriction (SR), defined as <7 h of sleep per night over extended periods, has become a common phenomenon [1–3]. Insufficient sleep in the workforce imposes a substantial economic impact through reducing productivity and increasing errors in decision making [4, 5]. While recent experimental studies have highlighted its impacts on various aspects of decision making, such as increased risk propensity [6–8], discounting of effort [9, 10], and heavier reliance on simplifying cognitive heuristics [11, 12], relatively little is known about how it influences the cognitive process by which individuals revise their beliefs over time in response to new, incoming evidence. Here, we refer to this process as *sequential updating*. In many real-world decision contexts, especially those marked by uncertainty and volatility, flexible decision making relies not just on interpreting current evidence, but also on integrating it with past experience to inform future choices. For example, a proprietary trader may initially place a long position based on earnings forecasts, but as the market provides newer economic indicators (e.g. inflation reports), they must adjust their stance, drawing on the new data but also how similar patterns played out in past trading cycles. Such decisions hinge on optimal integration of current evidence with accumulated knowledge. Given the prevalence of chronic SR, it is crucial to understand how it alters the cognitive underpinnings of sequential updating.

Sequential updating is often conceptualized within a Bayesian framework, wherein *prior* beliefs about the state of the environment are represented as probability distributions updated given new evidence (*likelihoods*) [13, 14]. This process involves mentally approximating the likelihood of competing hypotheses given evidence at hand, and integrating this with familiar, *prior* knowledge to generate *posterior* estimates. Importantly, these posteriors serve as priors for subsequent decisions, resulting in an iterative process of experiential learning and belief optimization. Alternatively, reinforcement learning models (e.g. Rescorla–Wagner model and Q-Learning) consider sequential updating as a trial-and-error process in which updating of learned reward values is driven by reward prediction errors, or the discrepancy between expected and received outcomes [15, 16]. These two frameworks imply distinct underlying cognitive mechanisms for sequential updating. Consequently, understanding how SR influences sequential updating requires arbitrating between these frameworks to reveal which processes are most vulnerable to its effects.

Our focus on sequential updating is motivated by prior work investigating the effects of SR on information integration under uncertainty [17, 18]. These studies utilize the Bayes Decisions Task, where participants are required to integrate two sources of Bayesian information on each trial: *prior* odds information that an event occurred and new evidence for that event representing Bayesian *likelihoods*. Across several experiments, we found SR reduced reliance on Bayesian *likelihoods*, while biasing participants toward leaning more heavily on prior odds [17, 18]. However, this task featured independent trials: outcomes of the participants’ choices had no bearing on future trials. Furthermore, no feedback was provided after choices were made, preventing participants from learning how future choices could be optimized. Since Bayesian posteriors, by definition, inform priors in future decisions, these features prevented the modeling of sequential updating dynamics, which is critical to understanding real-world decision making. To address these limitations, the present study employed a probabilistic reversal learning task (PRLT) where reward contingencies were probabilistic (i.e. responding correctly does not always guarantee reward), and these contingencies persisted across trials with a small chance of reversing. This setup requires participants to continuously engage in feedback-driven belief updating and adapt behavior dynamically in response to uncertainty.

Previously, Whitney et al. [19] employed a hybrid Go/No-Go reversal learning (GNGr) task, showing 62 h of total sleep deprivation (TSD) impaired both the initial learning and post-reversal adaptation. These deficits were accompanied by blunted skin conductance responses to feedback, indicating reduced sensitivity to outcome information. A follow-up study imposing 30.5 h TSD replicated this feedback-blunting effect with a GNGr variant modified to minimize working memory demands, suggesting these impairments persist even under shorter periods of extended wakefulness and reduced cognitive loads [20]. Notably, both studies reported sleep-deprived participants exhibited reduced ability to distinguish between Go and No-Go stimuli after reversals occurred, possibly indicating feedback was not effectively used to update stimulus–response mappings. Taken together, these findings indicate sleep loss disrupts feedback-based updating in volatile decision environments. However, the TSD protocols in both studies represent a distinct and acute form of sleep loss less commonly experienced than SR in the real world.

Where SR is concerned, two computational modeling studies may provide insights into how it affects sequential updating. Measuring risk propensity using the classic Iowa Gambling Task [21], Olson et al. [22] showed greater daytime sleepiness and recent sleep debt were associated with increased updating parameter values in the Expectancy Valence model [23], indicating participants relied more heavily on recent outcomes and incorporated less information from temporally distant feedback when sleepy. Additionally, and of more direct relevance to the present study, Gerhardsson et al. [24] examined the effects of SR using a probabilistic selection task, modeling performance using an asymmetric Rescorla–Wagner model where separate learning rates were estimated for positive (rewards) or negative (nonrewards) feedback. Results indicated SR selectively reduced sensitivity to negative feedback. These two studies offer a nuanced view of SR’s impact: SR disrupts sequential updating by biasing learning toward recent feedback at the expense of previously learned knowledge, with a further bias towards attenuated learning from negative feedback. However, our synthesis here should be interpreted with caution, given the studies differ in both task demands and model specifications.

It is also important to note limitations in sleep protocols employed by these two studies. First, Olson et al. [22] were not a direct investigation of SR, relying on self-reported daytime sleepiness and recent sleep durations to infer sleep debt in the absence of objective sleep monitoring methods (e.g. actigraphy and polysomnography). Second, Gerhardsson et al. [24] involved only two nights of 4 h sleep opportunities. In real-world contexts, many individuals experience more prolonged periods of SR due to ongoing demands from work and personal obligations. Prior research indicates the cognitive consequences of SR may be dose-dependent, with impairments compounding across consecutive nights [25]. This raises the question of how sequential updating may be affected under more prolonged periods of SR. The present study aimed to address this limitation through implementing an SR protocol involving seven consecutive nights of 5 h sleep opportunities.

To examine how SR alters the cognitive mechanisms underlying sequential updating, we applied computational modeling methods and compared three models grounded in reinforcement learning and Bayesian updating frameworks, plus a null model. As no studies to date have directly compared these models in the context of SR, we adopted an agnostic data-driven approach involving model comparison, without making an a priori commitment to which model would best fit the data. Aside from the novelty of the research question, this approach was also motivated by limitations in prior literature. Specifically, Olson et al. [22] and Gerhardsson et al. [24] each relied on a single cognitive process model, and while their findings offer valuable insights, individuals may use alternative strategies falling outside the scope of preferred cognitive frameworks in approaching a given cognitive task [26]. Thus, a formal model comparison approach would offer a principled method for evaluating which cognitive account offers the most plausible explanation for behavior, ensuring subsequent interpretations of SR-induced cognitive effects are guided by assumptions of the model best supported by the data. Given the cumulative evidence, though, we hypothesized SR would alter latent cognitive processes involved in sequential updating, specifically in model parameters reflecting sensitivity toward outcome feedback over time.

## Materials and Methods

### Participants

Our a priori analysis using G*Power 3.1 [27] for a repeated-measures ANOVA design (within-subjects; 2 groups × 2 measurements) indicated a sample size of 34 participants was required to detect a medium effect at α = .05 with 80% power. The present study included 36 healthy participants (M_age_ = 24.46, SD_age_ = 5.07, 25 females, 11 males). Prescreening eligibility criteria included: 18–39 years old, fluent in English, and maintaining a habitual sleep schedule with bedtime between 10 p.m. and 12 a.m., waketime between 6 a.m. and 9 a.m., and total sleep duration of 7–9 h. Participants were also required to have access to a personal computer or laptop with internet connectivity for remote cognitive testing. Participants were subsequently screened for history of serious medical or psychiatric conditions, familial risk of mood or psychotic disorders, self-reported sleep disorders, nicotine use (current or past), use of recreational drugs, or nonprescribed medications within the past three months, excessive daily caffeine intake (>400 mg/day), excessive alcohol use (>10 standard drinks/week or > 6 in a day), shift work within the past three months, or a body mass index falling outside a range of 18.5–30. Additional screening questionnaires were administered to identify factors potentially affecting sleep or cognitive performance. Table 1 provides a list of these questionnaires and their respective exclusion thresholds.

**Table 1:** Screening questionnaires and respective thresholds for exclusion

Questionnaire	Threshold for exclusion
Morningness–Eveningness Questionnaire [28]	Score between 22 and 25 or between 4 and 7, indicating extremely advanced or delayed sleep phase
Epworth Sleepiness Scale [29]	Score > 10, indicating excessive daytime sleepiness
STOP-Bang [30]	Score > 5, indicating moderate or severe risk of obstructive sleep apnea
Pittsburgh Sleep Quality Index [31]	Score > 5, indicating poor sleep quality
Patient Health Questionnaire [32]	Score > 5, indicating depressive symptoms
Beck Anxiety Inventory [33]	Score > 15, indicating anxiety symptoms
Alcohol Use Disorders Identification Test [34]	Score ≥ 8 for males or ≥ 6 for females, indicating potential alcohol consumption problem
Drug Use Disorders Identification Test [35]	Score ≥ 6 for males or ≥ 2 for females, indicating potential drug-related problem

### Design and procedure

The present study utilizes a within-subjects design. All participants were required to complete two sleep conditions: well-rested (WR), involving 9 h time-in-bed (TIB) for seven nights; and SR, involving seven nights of 5 h TIB. Condition order was counterbalanced across participants, and participants completed their respective protocols entirely at home. Sleep and wake times for WR were individualized based on self-reported habitual sleep schedules at screening. Because not all participants habitually obtained 9 h of sleep prior to study involvement, we ensured the prescribed 9 h sleep opportunity was compatible with their personal schedules. Where adjustments were necessary, prescribed bedtimes were kept within 1 h of participants’ habitual bedtimes to minimize circadian misalignment. For SR, we achieved the 5 h TIB by reducing sleep time equally in the evening and morning, relative to the midpoint of their assigned WR sleep period. Prior to each experimental week in both conditions, participants underwent a wash-in period consisting of 5 nights 8–9 h TIB. In total, each condition lasts 13 days and 12 nights. Adherence to prescribed sleep periods was assessed via wrist-worn actigraphy (Fitbit Charge 5 s or Alta HRs). Additionally, participants were required to complete daily sleep diaries and left voicemail recordings each night before bedtime and each morning upon waking.

**Figure 1:**
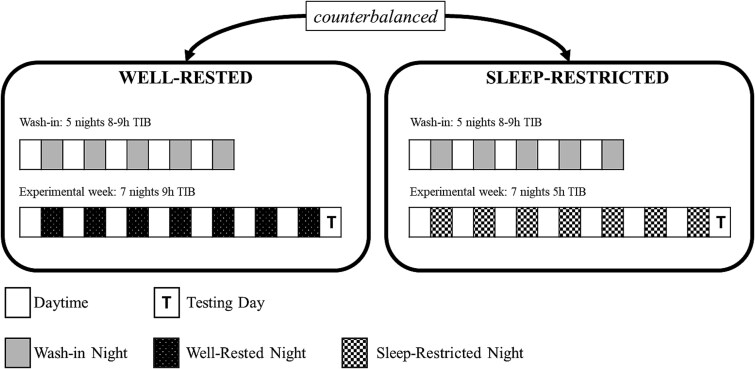
Study protocol*.* All participants completed WR and SR conditions in counterbalanced order. Both experimental sleep conditions were conducted entirely from home, and testing was conducted remotely on the morning of the 13^th^ day.

Participants were instructed to abstain from caffeine consumption for at least 12 h prior to cognitive testing on the 13^th^ day. Cognitive testing involved a decision-making battery, administered 2 h post-wake for WR, and 4 h post-wake for SR. This ensured testing was conducted at a consistent clock time across both conditions to maintain circadian alignment between conditions and provide additional time for the dissipation of cognitive deficits due to sleep inertia, which is typically more pronounced under SR [36, 37]. Within the battery, task order was kept consistent within each participant for both WR and SR, but counterbalanced across participants. Only the sequential updating task is reported here. A visual representation of the study protocol is illustrated in Figure 1.

By default, participants completed the two conditions back-to-back: following completion of testing on the 13^th^ day of the first assigned condition, they commenced the wash-in period for the second condition that same night. In cases where participants could not complete both conditions back-to-back due to scheduling conflicts, gaps of nonparticipation between conditions were permitted, provided these did not interfere with study logistics.

### Materials

#### Karolinska sleepiness scale

To ensure our sleep manipulations were effective, we administered the Karolinska sleepiness scale (KSS) as a measure of subjective sleepiness prior to each decision-making battery [38]. The KSS consists of a single item where participants rated their sleepiness levels on a 9-point scale, with 1 = “extremely alert” and 9 = “extremely sleepy.” Previous research indicates KSS scores are correlated with behavioral and electroencephalographic indicators of sleepiness [39–41].

#### Probabilistic reversal learning task

**Figure 2:**
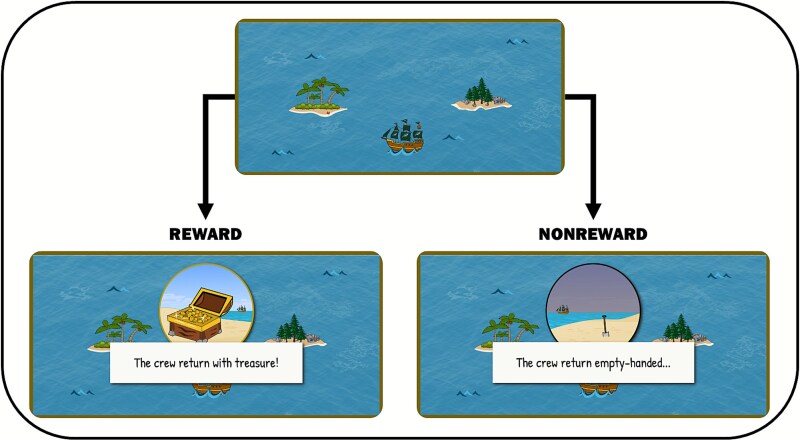
Example of a trial on the probabilistic reversal learning task on each trial, one of the two islands provide a 70% chance of reward, while the other, 30%. Participants are informed these reward contingencies persist across trials, with a 10% chance of reversing on each new trial.

To assess sequential updating, we administered a gamified probabilistic reversal learning paradigm (the PRLT) as one of three tasks in a broader decision-making battery. Previous research demonstrates this paradigm offers a valid and sensitive measure of cognitive flexibility and sequential updating, requiring individuals to adapt their behavior in response to changing reward contingencies by continuously updating stimulus–reward associations across multiple reversals [42–45]. On each of the 150 active trials, participants are shown two islands in the middle of a computer screen and required to choose between “visiting” each island. They are told (1) one island will yield reward 70% of the time, while the other yields reward 30% of the time, and (2) the probability of reward associated with each island persists from trial to trial, but there is a 10% chance these contingencies reverse on each new trial. Participants are not informed when reversals occur. To maximize the amount of reward earned, participants should repeatedly sample each island across several trials to determine which island is more likely to yield reward and adjust accordingly when reversals occur. At the end of each testing session, the number of rewarded trials was converted into bonus monetary reward in addition to the original study compensation. Figure 2 shows an example of a trial on the PRLT.

To ensure comprehension of task instructions, participants were administered a 4-item comprehension quiz at the start of the task. Corrective feedback was shown if any question was incorrectly answered. Additionally, participants underwent a 25-trial practice phase prior to the active trials.

Reversals during the practice phase were discreetly set to occur every 10 trials, allowing participants two opportunities to encounter reversals. For trials after the practice phase, reversals had a 10% chance of occurrence on each trial in accordance with task instructions, subject to a discreet constraint that a minimum of 5 and a maximum of 25 trials separated each reversal. These limits ensured opportunities for learning reward contingencies were appropriate in length and comparable across participants.

### Data handling and analyses

All data analyses were conducted in R ver. 4.3.0 [46]. Where models utilized maximum-likelihood estimation, significance was set at α = .05. Of the initial 36 participants, two failed to complete their SR conditions due to nonadherence with their sleep schedule. Of the 34 participants who successfully completed both conditions, 16 completed both conditions back-to-back, 16 had a nonparticipation gap ranging 1–7 days, and two had gaps longer than a week (11 and 16 days). One participant encountered server disruptions during remote testing, and hence their WR dataset was lost, resulting in a final total of 33 complete datasets. The same participant also failed to complete the KSS prior to both testing sessions. As our modeling approaches are robust to missing data, we retained the remaining available dataset for each of the three affected participants.

#### Subjective sleepiness

To investigate whether SR significantly influenced subjective sleepiness, we fitted a random-intercept linear mixed model regressing KSS scores on a factored *condition* variable (WR/SR). As assumption checks indicated a significant Levene’s test, we applied log transformations to KSS scores to maintain homogeneity of residual variance.

#### Probabilistic reversal learning task

On average, participants encountered 6.88 (SD = .65) reversals in WR with 10.22 (SD = .57) trials before reversals, and 6.97 (SD = .80) reversals in SR with 10.07 (SD = .75) trials before reversals. A two-sample *t*-test indicates this difference was not significant (*p*=.608).

#### Conscientious responder check

Prior to data analyses, we screened the PRLT data for nonconscientious responses by first identifying session data with total scores (no. of trials rewarded) in the bottom 10^th^ percentile, then inspecting these data both visually and algorithmically for straightlining. Specifically, our straightlining detection algorithm identified when participants made at least 15 consecutive identical choices (15 as a midpoint between min. and max. possible *n* trials before reversals). In these subsets of ≥15 trials, the corresponding session data would be flagged for straightlining if the participant was not rewarded on more than half the trials, indicating they failed to adjust their choices even when unrewarded most of the time. Based on these criteria, two WR datasets were further identified and dropped.

#### Trial accuracy

We fitted a random-intercept logistic mixed model to examine differences in trial accuracy attributable to *condition*. The outcome *ptCorrect* was created by matching participant choices on each trial with the current “correct” choice (i.e. the option currently yielding reward at a 70% rate), denoted 1 if correct, and 0 otherwise. To control for potential learning effects on the PRLT, we also included a factored *session* predictor, denoting whether participants were performing the PRLT for the first or second time, irrespective of *condition*.

#### Computational modeling

To determine the best cognitive account of participant performance on the PRLT, we fitted and compared three cognitive process models to the pooled dataset comprising both WR and SR data: the standard Rescorla–Wagner model [16] with a single learning rate parameter (i.e. irrespective of feedback type); an asymmetric variant of the Rescorla–Wagner with two learning rate parameters (one for reward feedback and one for nonreward feedback); and a Bayesian Precision Weighting model. All models were implemented within a Bayesian hierarchical framework using the *cmdstanr* package in R [47], and estimated via Markov Chain Monte Carlo (MCMC) sampling. Each model was run with four chains, 2500 warm-up iterations, and 6000 post-warmup iterations per chain. Weakly regularizing priors were used throughout. Model comparisons were based on Watanabe-Akaike Information Criterion (WAIC), an estimate of out-of-sample prediction accuracy for Bayesian models [48].

For both Rescorla–Wagner models, let ${Q}_t^A$ represent the expected value of selecting island *A*, before selecting it on trial *t*. For trial *t =* 1, ${Q}_t^A={Q}_t^B=0.5$, reflecting initial complete uncertainty about the value of each island.


(1) \begin{equation*} {Q}_{t+1}^A={Q}_t^A+\eta \left({R}_t-{Q}_t^A\right) \end{equation*}

Equation (1) provides a delta updating rule to compute the expected value of selecting island *A* on the next trial *t + 1*, assuming island *A* is selected. On the PRLT, ${R}_t=1$ when participants are rewarded, and ${R}_t=0$ otherwise (nonreward). The model assumes individuals integrate the difference between the outcome ${R}_t$and ${Q}_t^A$, with subjective sensitivity to feedback determined by individual “learning rates” $(\eta, \kern0.5em 0\le \eta \le 1;$ higher values = increased sensitivity). If island *A* remains unselected, ${Q}_{t+1}^A={Q}_t^A$.

The asymmetric variant of the Rescorla–Wagner model differs from the base version in its use of two distinct learning rate parameters, depending on whether the decision outcome was positive or negative. On the PRLT, this is conceptualized as whether participants received reward $\left({R}_t=1\right)$ or nonreward $\left({R}_t=0\right)$following their choices:


(2) \begin{equation*} {Q}_{t+1}^A=\left\{\begin{array}{c}{Q}_t^A+{\eta}^{+}\left({R}_t-{Q}_t^A\right)\\{}{Q}_t^A+{\eta}^{-}\left({R}_t-{Q}_t^A\right)\end{array}\genfrac{}{}{0pt}{}{if\ {R}_t=1}{if\ {R}_t=0}\right. \end{equation*}

Similarly, ${\eta}^{+}$ and ${\eta}^{-}$ range 0 to 1, and higher values indicate increased rate of learning, or sensitivity toward the corresponding type of feedback.

Based on the expected value of choosing each island, the probability of choosing island *A* from two options on trial *t* is given by the softmax choice rule [49]:


(3) \begin{equation*} pr{\left(\mathrm{choose}\ A\right)}_t=\frac{e^{\beta \bullet{Q}_t^A}}{\sum_{i=1}^2{e}^{\beta \bullet{Q}_t^i}} \end{equation*}

Here, the inverse temperature parameter $\beta$(ranging 0 to $\infty )$captures the degree of consistency in the individual’s choice patterns. Higher values of $\beta$ indicate a higher degree of alignment between choices and cognitive assumptions of each model (i.e. an individual is more likely to choose the island they believe will garner a reward), while lower values indicate more stochasticity ($\beta =0$ indicates completely random choice). Overall, these mathematical formalizations imply choices with a more consistent rate of yielding reward are reinforced, and thus, more likely to be repeated.

In contrast to reinforcement learning models, the Bayesian Precision Weighting model assumes individuals maintain and update explicit probabilistic beliefs about which island is the current “good” option (i.e. “island *A* is now the one with 70% chance of reward”). These beliefs are updated in a Bayesian fashion, where prior beliefs are combined with likelihood information inherent in trial feedback. Importantly, this also implies individuals are constantly cognizant of the chance at which a reversal might occur (${p}_{\mathrm{switch}}=.10$).

Let ${b}_t^A$ represent the subjective prior belief that island *A* is the good option on trial *t*. Equation (4A) and (4B) provides the Bayesian updating rule to calculate the posterior belief of island *A* being more advantageous.


(4A) \begin{equation*} {\tilde{b}}_{t+1}^A={\omega}_{\mathrm{prior}}\bullet{b}_t^A+{\omega}_{\mathrm{evid}}\bullet{\mathcal{L}}_t^A \end{equation*}

where


(4B) \begin{equation*} {\mathcal{L}}_t^A=p\left({S}_t=A|{R}_t,{a}_t=A\right) \end{equation*}

In Equation (4A), ${\mathcal{L}}_t^A$is the Bayesian *likelihood* term, or the inferred probability of the current good option being island A *solely* from feedback after choosing A (Equation 4B). Since the chance of reward from choosing the good option remains constant across trials, it is always the case that ${\mathcal{L}}_t^A=.70$ if choosing A yields a reward, and .30 otherwise. ${\omega}_{\mathrm{prior}}$ and ${\omega}_{\mathrm{evid}}$ are weighting parameters quantifying reliance on Bayesian priors and likelihoods, respectively. ${\omega}_{\mathrm{prior}}$ is bounded between 0 and 1, where higher values indicate more reliance on the corresponding source of information. Additionally, the model assumes these weights are inversely proportional (i.e. ${\omega}_{\mathrm{evid}}=1-{\omega}_{\mathrm{prior}}$).

Next, the posterior belief ${\tilde{b}}_{t+1}^A$ is adjusted via hazard-based belief mixing to reflect uncertainty about the good option due to the 10% chance of reversal on the next trial. This yields the prior belief for trial *t + 1*:


(5) \begin{equation*} {b}_{t+1}^A=\left(1-{p}_{\mathrm{switch}}\right)\bullet{\tilde{b}}_{t+1}^A+{p}_{\mathrm{switch}}\bullet \left(1-{\tilde{b}}_{t+1}^A\right) \end{equation*}

The softmax choice rule for the Bayesian Precision Weighting model likewise includes an inverse temperature $\beta$ parameter capturing choice consistency:


(6) \begin{equation*} pr{\left(\mathrm{choose}\ A\right)}_t=\frac{e^{\beta \bullet{b}_t^A}}{\sum_{i=1}^2{e}^{\beta \bullet{b}_t^i}} \end{equation*}

To examine SR-induced shifts in latent decision mechanisms, we introduced offsets for each base parameter in all models. As an example, the effective value of $\eta$ in each condition for the Rescorla–Wagner learning rate is modeled as:


(7) \begin{equation*} {\eta}_{\mathrm{eff}}^i=\phi \left({\eta}_{\mathrm{WR}}^i+\mathrm{condition}\bullet \Delta{\eta}_{\mathrm{SR}}^i\right) \end{equation*}

where ${\eta}_{\mathrm{WR}}^i\in \mathbb{R}$ is the learning rate estimate for participant *i* during WR on the untransformed real scale, and condition *=* 1 for SR, and 0 otherwise. The term $\Delta{\eta}_{\mathrm{SR}}^i\in \mathbb{R}$ represents the untransformed offset parameter capturing shifts in learning rate under SR.

Finally, we also fitted a null model where participants were assumed to select each option based on a fixed individual-level probability, estimated from their respective overall distributions across trials. These individual choice probabilities were allowed to vary by condition. Importantly, the null model included no trial-by-trial feedback-based updating process, treating choice on the PRLT as a stochastic process. By including this in model comparisons, we were able to test the three cognitive process models against the null hypothesis that participants did not engage in sequential updating.

### Pilot testing: learning effects

To assess potential learning effects on the PRLT, we previously conducted pilot testing with 30 participants recruited via Prolific (aged 18–39 years). Pilot participants completed the PRLT twice, two weeks apart, without any sleep manipulation. This interval approximated the gap between conditions in the main study when completed back-to-back. The pilot data were analyzed using the same procedures described above, and results are included in the Supplementary materials. Briefly, no evidence of learning effects was observed in either trial accuracy or component free parameters of the fitted models, hence providing greater confidence the effects reported below are likely attributable to SR rather than repeated testing.

## Results

### Actigraphy and total sleep times

Altogether, seven nights of actigraphy data (two from one participant, one each from five participants) were missing due to improper wearing of the Fibtit device or insufficient prior battery charging by the participant. In these cases, researchers verified protocol adherence by checking the timestamps of voicemail recordings and sleep diaries. Actigraphy data indicated for wash-in nights, participants logged average total sleep times (TSTs) of 8.02 h (SD = .45) for WR and 7.83 h (SD = .61) for SR. For the seven experimental nights in each condition, participants logged average TSTs of 7.96 h (SD = .47) for WR and 4.89 h (SD = .39) for SR. A random-intercept linear mixed model fitted to the pooled WR and SR data (excluding wash-in periods) indicated a significant effect of SR (*p*<.001), indicating participants in the SR condition obtained significantly less sleep, relative to WR.

### Subjective sleepiness

Mean raw KSS scores were 2.63 (SD = 1.24) for WR, and 5.03 (SD = 2.14) for SR. There was a significant effect of *condition* on log-transformed KSS scores, indicating participants felt significantly sleepier during SR (*p*<.001). Model estimates are presented in the Supplementary materials.

### Trial accuracy

There were no significant effects of *condition* or *session*, indicating neither SR nor repeated testing influenced the number of times participants chose the more advantageous option on the PRLT (see Supplementary materials).

### Computational modeling

All models showed good convergence, with $\hat{R}$ values close to 1 and no divergent transitions during sampling. Model WAICs indicated the null model provided the worst fit (Table 2), indicating that participants’ behavior on the PRLT were consistent with sequential updating. The Asymmetric Rescorla–Wagner model provided the best fit of the data, and thus we only report parameter estimates of this model here. For parameter estimates from Rescorla–Wagner and Bayesian Precision Weighting models, as well as details on posterior predictive checks for each model, we refer the reader to the Supplementary materials.

**Table 2:** Computational modeling: model comparisons

Model	ΔWAIC	ΔWAIC (SE)
Null model	7133.8	101.0
Rescorla–Wagner	71.0	20.0
Asymmetric Rescorla–Wagner	0	0
Bayesian precision weighting	645.8	83.8

WR, well-rested; SR,  sleep-restricted; SE,  standard error of ΔWAIC.

WAIC values reported here are based on models fit to the pooled dataset (both WR and SR). Lower WAIC values indicate better fit, and ΔWAIC values presented here indicate the difference between the best fit (ΔWAIC = 0) and the corresponding model.

**Figure 3:**
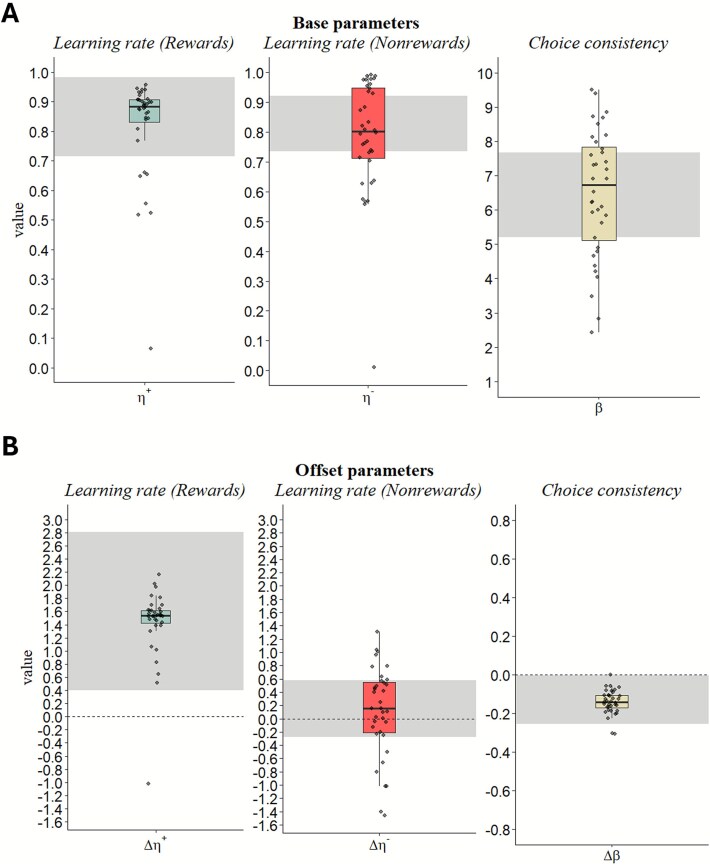
Parameter estimates for asymmetric Rescorla–Wagner model*.* Shaded bands along the *y*-axis indicate 95% Bayesian credible intervals (CI). Individual-level estimates (represented by scatter points) are informed by group-level distributions under a Bayesian hierarchical framework. (A) Base model parameter estimates when WR. (B) Offset parameter estimates (untransformed), representing changes in the corresponding baseline parameters when sleep-restricted. Effects of SR are interpreted as credible if the 95% CI of offset parameters excludes zero.

For parameter estimates, we considered the effect of SR to be credible if the 95% credible intervals for the corresponding offset parameter excluded 0. As such, we also report offset parameter values on the untransformed real scale, while base parameters are reported with their respective transformations applied (Table 3 and Figure 3). Based on this criterion, our results revealed a credible increase in the reward learning rate parameter ($\Delta{\eta}^{+}>0)$ and decrease in choice consistency ($\Delta \beta <0$) under SR, indicating SR resulted in increased sensitivity to reward feedback (but not negative feedback), and more stochastic, less goal-directed choices on the PRLT.

**Table 3:** Median and 95% highest density interval of Asymmetric Rescorla–Wagner model estimates

Parameters	Median	95% Bayesian CI	Convergence statistics	
			$\hat{R}$	ESS
Base				
Learning Rate for Rewards (${\eta}^{+})$	.86	[.72, .98]	1.00	6895
Learning Rate for Nonrewards (${\eta}^{-})$	.84	[.73, 92]	1.00	3165
Choice Consistency ($\beta)$	6.38	[5.20, 7.67]	1.01	808
Offsets				
Learning rate for rewards ($\Delta{\eta}^{+})$	**1.46**	**[.41, 2.77]**	**1.00**	**1062**
Learning rate for nonrewards ($\Delta{\eta}^{-}$)	.11	[−.28, .59]	1.00	3303
Choice consistency ($\Delta$)	**−.14**	**[−.26, −.003]**	**1.01**	**579**

CI, credible interval; ESS, effective sample size.

Parameter estimates were derived by fitting the model to the pooled dataset comprising both WR and SR conditions. Offset parameter values are reported on the untransformed real scale, and SR effects are deemed credible if 95% Bayesian CIs for these parameter estimates exclude 0 (**bolded**).

## Discussion

The present study investigated how SR affects the cognitive mechanisms underlying sequential updating in uncertain decision environments. Motivated by prior work, we hypothesized SR would alter sequential updating by disrupting sensitivity to outcome feedback, though we remained agnostic as to whether these disruptions would be best captured by a Bayesian updating or reinforcement learning framework. Model comparisons indicated the asymmetric Rescorla–Wagner model best described participants’ behavior on the PRLT, indicating choice behavior followed a reinforcement learning process in which participants learned at different weights for positive versus negative feedback. In line with our broader hypothesis, results revealed credible SR-related increases in reward sensitivity and choice stochasticity, indicating sleep-restricted individuals were more strongly influenced by positive outcomes while exhibiting reduced consistency in their decision strategies.

### Sequential updating on the PRLT reflects asymmetric sensitivity to feedback

In model comparisons, the Asymmetric Rescorla–Wagner model outperformed both the symmetric Rescorla–Wagner model and the Bayesian Precision Weighting model. Neurophysiological evidence provides support for natural asymmetry in how feedback is processed: D1 receptor pathways are thought to facilitate reward-driven action selection, while D2 receptor pathways inhibit responses given negative outcomes [50]. Furthermore, dopaminergic firing encodes positive prediction errors more robustly than negative ones [51]. On tasks like the PRLT, asymmetric learning may enhance sequential updating through filtering out noisy feedback and accentuating feedback information indicative of changes in a volatile decision environment (e.g. protracted sequences of nonrewards where reward was expected, indicating a change in the favorable island) [52, 53].

Additionally, while Bayesian updating models offer an elegant account of “ideal,” rational learning, they are mentally difficult to implement [54–56]. A core component of our Bayesian Precision Weighting model is its inclusion of a 10% hazard rate, representing the chance of a reversal on the PRLT. Although this structure was explicitly stated in task instructions, the actual occurrence of reversals was never explicitly signaled to participants during the task, rendering them unobservable. Consequently, participants were required to detect reversals implicitly through feedback information rather than via direct cues. Under these conditions, Bayesian updating becomes a cognitively taxing process [57]: successful implementation would require constantly monitoring the precision of one’s beliefs based on the likelihood of a reversal, in addition to tracking probabilistic feedback. These cognitive demands, which are even more aversive under SR [10], may lead participants to fall back on simpler feedback-driven strategies. Our interpretation here aligns with previous findings showing even minor increases in structural uncertainty can impair the fits of Bayesian updating models, prompting reliance on more tractable reinforcement learning processes [57, 58].

### SR increases sensitivity to rewards

Crucially, on the surface, our results seem to diverge from those of Gerhardsson et al. [24], who reported a selective reduction in sensitivity toward negative outcomes despite also implementing a similar asymmetric variant of the Rescorla–Wagner model. However, these findings are not necessarily in direct contrast. As shown in Niv et al. [59], reinforcement learning models with asymmetric learning rates for positive and negative outcomes capture how the relative difference between reward and nonreward sensitivity shapes behavior. Specifically, it is the *asymmetry* between these learning rates, rather than their absolute values, that shape risk-sensitive profiles. An increase in sensitivity to rewards or a decrease in sensitivity to nonrewards can each amplify this asymmetry and lead to comparable behavioral outcomes, implying both phenomena may be two sides of the same underlying process. Furthermore, methodological differences across both studies may have contributed to differences in findings.

One important distinction lies in the cognitive demands imposed by the respective task paradigms. Specifically, our PRLT featured multiple undisclosed reversals in reward contingencies over 150 trials, placing sustained demands on exploratory information seeking behavior to continually probe for the more advantageous option. In contrast, Gerhardsson et al. [24] modeled learning on a shorter 60-trial learning phase within a probabilistic selection task, during which reward contingencies remained static. This more predictable environment may have encouraged individuals to settle into more exploitative choice patterns (i.e. repeatedly picking the advantageous option) on later trials based on previously learned reward contingencies, reducing the need for ongoing exploration. Thus, negative feedback would be less likely to alter choice behavior, and learning rates from negative feedback would necessarily decrease. Prior evidence from Daw et al. [60] showed decision making on a task requiring both exploration and exploitation engaged both the anterior frontopolar cortex, a region associated with coordinating between competing goals [61, 62]; and the medial orbitofrontal cortex, involved in tracking and updating the relative value of rewarding outcomes [63]. This implies under conditions where continuous exploration is required, reward signals become more salient as they provide crucial evidence indicating the more advantageous choice. This may have motivated our participants toward reward acquisition.

A second distinction concerns differences in the duration of SR imposed. While the SR period in Gerhardsson et al. [24] lasted two nights, we imposed a longer period involving seven nights. Previous evidence by Van Dongen et al. [25] demonstrated a dose–response effect of SR, where cognitive processes, including those relevant to sequential updating (e.g. sustained attention, processing speed), progressively worsened over a 14-day period of 4 h or 6 h TIB. Hence, a higher dose of SR may account for the broader pattern of cognitive changes we observed, particularly the additional effect on choice stochasticity.

Increases in reward sensitivity under SR may reflect underlying changes in dopaminergic activity in the brain. Past neuroimaging studies suggest sleep loss reduces D2 and D3 receptor availability in the ventral striatum, causing D1 receptors to become overstimulated by presynaptic dopamine [64, 65]. This neurochemical imbalance may suppress behavioral inhibition and risk assessment while heightening reward-seeking and risk-taking. Other behavioral evidence provide further support: one night of TSD shifted individuals’ economic preferences from avoiding losses to pursuing gains [66]; habitual nappers prevented from taking their daytime naps exhibited a greater preference for immediate, smaller rewards over delayed, larger rewards [67]; and five nights of 6.5 h TIB increased the subjective appeal and value of food rewards in adolescents [68]. Through computational cognitive modeling, we quantify a similar effect of SR on the PRLT, where SR may have disrupted longer-term strategies based on assessing outcome values associated with both options, and instead biased decision making toward reward-chasing strategies.

Alternatively, motivational mechanisms may also contribute to increased reward sensitivity under SR. Evidence from the effort discounting literature shows individuals experiencing sleep loss are more likely to forgo rewards requiring greater effort, opting instead for smaller, easier-to-obtain alternatives [9, 69, 70]. Notably, Jurgelis et al. [10] reported greater effort discounting under SR even when participants performed comparably across varying difficulty levels of a cognitive task, suggesting an increased aversion to cognitive effort underlies the effects of SR, rather than a mere reduction in perceived ability to perform optimally. On the PRLT, maintaining and updating value representations for both options across successive trials may be cognitively effortful. Sleep-restricted participants may therefore adopt a less demanding strategy, relying on the most recent rewarding outcome as a heuristic to guide choice behavior, adopting a heuristic-based strategy of “following the reward trail” over sustained representations of long-term stimulus–reward mappings.

Aside from methodological differences in how sleep loss was induced, this motivational account also provides a plausible framework for reconciling our findings with previous studies demonstrating TSD-induced feedback-blunting effects on reversal learning paradigms [19, 20]. Under acute TSD, feedback blunting manifests as a reduction in the salience and efficacy of outcome feedback, as reflected in diminished skin conductance responses and impaired reversal learning [19, 20]. Our SR data possibly reveal a valence-specific expression of this phenomenon. Specifically, reliance on lower-effort strategies prioritizing immediately rewarding cues may reduce the salience of reward histories over consecutive trials, thereby diminishing the affective impact of overall trial feedback. Hence, the “reward-chasing” profile observed here suggests a selective form of feedback blunting, where chronic SR reduces cognitive effort invested in integrating feedback over time, while preserving responsiveness to positive reinforcement.

### SR increases choice stochasticity

Our findings also lend to a growing body of evidence showing sleep loss increases choice stochasticity (i.e. randomness) during decision making. Several studies have also observed similar increases in stochasticity attributable to TSD across various cognitive process models, applied to a variety of tasks assessing effort-based decisions and risk propensity [9, 70, 71]. Notably, our recent work [26] modeling previously published data [6] showed 51 h of continuous wakefulness increased choice stochasticity on the Iowa Gambling Task [21], indicating an increase in genuinely “noisy” choices rather than goal-directed exploration. These findings may reflect converging effects of sleep loss on underlying neural mechanisms. In addition to its effects on dopaminergic activity in the ventral striatum [64, 65], sleep loss reduces the activation of frontoparietal networks critical for top-down attentional control, potentially impairing the ability to monitor, and implement consistent cognitive strategies over time [72–74]. Moreover, it also attenuates activity in the anterior cingulate cortex, bilateral insula, and putamen [75], areas implicated in the process of integrating feedback with learned knowledge, encoding prediction errors and tracking stimulus–reward mappings [76–78]. Taken together, SR-induced impairments in these neural substrates of cognitive control and value representation may contribute to more erratic, less goal-driven responding, manifesting as increased choice stochasticity on the PRLT.

### Limitations and other considerations

There are limitations to consider in interpreting results of the present study. Although data from 36 participants were included, complete datasets for *both* sleep conditions were available for only 33 participants due to aforementioned nonadherence and technical issues. Our a priori power analysis indicated a required sample of 34, and hence the number of complete datasets was only marginally below this threshold. While this may pose sample size concerns for models using frequentist methods (i.e. KSS and trial accuracy analyses), we note Bayesian hierarchical models are more robust to such constraints. Through the use of partial pooling and weakly informative priors, our computational models provide stable parameter estimates, thereby mitigating the impact of missing data and modest sample size on inferential reliability [79–81]. Secondly, our sample primarily consists of healthy young adults with stable sleep patterns and no reported sleep or psychiatric disorders. Consequently, our findings are most directly relevant to healthy, nonclinical young adults with comparable demographic profiles. Given the diversity of individuals in professions where chronic SR is common, future research may attempt to investigate whether these effects generalize to older adults or populations with mood, sleep, or cognitive disorders.

Thirdly, our protocol was conducted entirely in participants’ homes. Hence, we were unable to ensure control for factors potentially influencing sleep quality and/or cognitive performance, such as environmental lighting [82] or potential third-party aid during cognitive testing. The at-home design meant we were unable to strictly enforce caffeine abstention during the 12 h period prior to cognitive testing, despite providing participants explicit instructions to do so. Notably, however, the observed effects on reward sensitivity and choice consistency occurred even with the possibility of caffeine consumption by participants during this period [83]. Where feasible, future research should implement in-lab protocols to allow for tighter experimental control over these extraneous variables, ensuring greater consistency in sleep conditions and cognitive testing environments.

Furthermore, although we monitored participants’ sleep opportunities via wrist-worn actigraphy and subjective data from sleep diaries and voicemail call-ins, we did not assess participants’ sleep architecture. Studies have shown Fitbit models to be sensitive in detecting broader sleep–wake patterns, but less so in providing precise quantification of sleep architecture [84–86]. Prior research implicates rapid eye movement (REM) and slow-wave sleep in risky decision making [87] and in core cognitive processes relevant to flexible decision making, including working memory and vigilant attention [88–90]. Hence, it is entirely possible for observed differences in PRLT performance to arise from changes in sleep architecture, especially considering our SR protocol curtailed the final 2 h of sleep when REM is most prevalent [91]. A more detailed understanding of sleep stage contributions to altered sequential updating under SR may be achieved through polysomnography and/or targeted sleep stage disruptions.

Lastly, though our results revealed no significant differences in trial accuracy on the PRLT between conditions, we caution against interpreting this as a benign effect of SR. The probabilistic nature of reward on the task, in addition to hidden reversals, introduces uncertainty around whether errors reflects knowledge of the advantageous option or exploration due to information-seeking needs [92]. Because the more advantageous option yields reward only 70% of the time, even “correct” choices can result in protracted sequences of nonreward feedback, making it difficult for participants to infer and monitor accuracy. Thus, trial accuracy is often a noisy and misleading indicator of decision-making performance in probabilistic paradigms. Our computational modeling results highlight the importance of investigating latent cognitive mechanisms underlying choice behavior, revealing meaningful effects of SR undetectable through surface-level metrics.

To improve sensitivity of accuracy metrics in future designs, several modifications to the probabilistic reversal learning paradigm may be considered. First, allowing reversal probabilities to shift across trials may allow researchers to better isolate the effects of volatility in the task environment from those attributable to sleep loss. Second, pseudo-randomized reward sequences can be pregenerated to ensure matched reward histories across participants, reducing variability in accuracy attributable to sampling noise and improving comparability between sleep conditions. Finally, collecting confidence ratings at fixed intervals may also help clarify whether errors reflect exploration due to uncertainty, or genuine impairment in sequential updating. Together, these refinements would help to retain relevance to real-world decision contexts offered by probabilistic paradigms, while offering improved sensitivity to potential decision-making errors arising from SR.

### Real-world implications

Our findings carry implications for real-world decision contexts, particularly those requiring the learning of optimal strategies over repeated decision iterations. In these settings, SR-induced hypersensitivity toward rewards may bias individuals toward premature judgments based on early positive outcomes, instead of gradual accumulation and weighing of evidence. Consequently, this may prompt risk-laden decisions in high-stakes, uncertain environments. For example, a proprietary trader may overreact to a single profitable trade by over-allocating capital to the same financial instrument, instead of monitoring broader market indicators. Alternatively, a casino patron may be more inclined to continue gambling in response to intermittent small wins despite its known risks. Moreover, SR-induced increases in choice stochasticity may result in haphazard and inconsistent decision making. This may carry serious consequences in high-stakes professional domains where reliable, evidence-based decision making is crucial, such as medicine, air traffic control, or law enforcement. Overall, the present findings underscore the need for targeted interventions in occupations prone to chronic SR, such as implementing relevant cognitive training programs, providing psychoeducation on the specific cognitive vulnerabilities associated with sleep loss, and introducing standardized scaffolding of operating procedures to help maintain decision consistency under sleep-restricted conditions.

## Supplementary Material

SEQ_suppmat_R1_zsaf354

## Data Availability

Data and *R s*cripts pertaining to reanalyses in this paper can be found at: https://github.com/jlim0063/seq_updating_analyses.
