# Supplementary material for: Sleep restriction increases reward sensitivity during sequential updating
Source: Sleep. 2025 Nov 6;49(3):zsaf354. doi: 10.1093/sleep/zsaf354 (PMC13017762; doi:10.1093/sleep/zsaf354)

Sleep Restriction Increases Reward Sensitivity during Sequential Updating:

**Supplementary Materials**

Jeryl Y L Lim^1,2^, Daniel Bennett^1,3^, Sean P A Drummond^1,2^

Institutional Affiliation(s):

^1^School of Psychological Sciences, Monash University, Melbourne, Victoria, Australia

^2^Turner Institute for Brain and Mental Health, Monash University, Melbourne, Victoria, Australia

^3^Melbourne School of Psychological Sciences, The University of Melbourne, Melbourne, Victoria, Australia

Corresponding Author(s):

Jeryl Lim (Jeryl.lim@monash.edu)
Sean Drummond (sean.drummond@monash.edu)

# Pilot Testing: Learning Effects on the Probabilistic Reversal Learning Task

In designing the PRLT, we anticipated the possibility of learning effects when administering the task over repeated sessions. Hence, we conducted pilot testing where 30 Australian participants (also 18-39 years old) were recruited on Prolific and administered the PRLT twice. Testing sessions were two weeks apart to mimic the gap between testing sessions in the actual study, assuming both experimental conditions were done back-to-back.

Firstly, to investigate changes in trial accuracy, we fitted a random-intercept linear mixed model, regressing *ptCorrect*, a binary variable denoting whether participants picked the more advantageous option on each trial, on a factored *session* predictor (1st or 2nd time attempt on the PRLT). Participants in the pilot phase achieved 67.28% (SD = 8.08) accuracy in the first session, and 68.17% (SD = 9.49) in the second session. No significant effect of *session* was present (*p* = *.387*), indicating trial accuracy on the PRLT was unaffected by repeated testing.

Secondly, we computationally implemented the two Rescorla-Wagner variants and the Bayesian Precision Weighting model to assess the sensitivity of their component parameters to repeated testing. Similar to analyses reported in the main text, “offset” parameters were included to capture deviations in their corresponding base parameters. For the pilot data, offsets were specified for *session* = 2, allowing us to quantify deviations in base parameters resulting from repeated testing.

Each model was implemented within a Bayesian hierarchical framework using Hamiltonian Monte Carlo sampling, with 4 chains, 1000 warmup iterations and 4000 post-warmup iterations per chain. Model comparisons using Watanabe-Akaike Information Criterion (WAIC) indicated the pilot data was also best described by the Asymmetric Rescorla-Wagner model (Table S1). Across all three models, none of the offset parameters showed evidence of repeated testing, suggesting the latent cognitive mechanisms captured were stable across testing sessions (Table S2). This provides greater confidence in attributing parameter changes observed within our main analyses to SR, rather than task familiarity.

Table S1

*Computational modelling on Pilot Data: Model comparisons*

| Model | ΔWAIC | ΔWAIC (SE) |
| --- | --- | --- |
| Rescorla-Wagner | 62.8 | 14.6 |
| Asymmetric Rescorla-Wagner | 0 | 0 |
| Bayesian Precision Weighting | 871.2 | 70.0 |

*Note.* WR = Well-Rested. SR = Sleep-Restricted. SE = standard error of ΔWAIC. WAIC values are presented on a deviance scale, and lower numbers indicate better fit.

Table S2

*Model parameter estimates for Pilot Data*

| Parameter | Median | 95% CI | Convergence Statistics | |
| --- | --- | --- | --- | --- |
|  |  |  | $\hat{R}$ | ESS |
| *Rescorla-Wagner* | | | | |
| Base |  |  |  |  |
| Learning Rate^§^ | .64 | [.15, .96] | 1.00 | 14455 |
| Choice Consistency^†^ | 9.35 | [0.91, 24.64] | 1.00 | 15883 |
| Offset (*session* = 2 deviation) |  |  |  |  |
| Learning Rate* | 0.36 | [-1.06, 1.78] | 1.00 | 14454 |
| Choice Consistency* | -0.48 | [-1.88, 0.90] | 1.00 | 15890 |
| *Asymmetric Rescorla-Wagner* | | | | |
| Base |  |  |  |  |
| Reward Learning Rate^§^ | .88 | [.37, 1.00] | 1.00 | 14189 |
| Nonreward Learning Rate^§^ | .62 | [.13, .95] | 1.00 | 13131 |
| Choice Consistency^†^ | 9.38 | [0.95, 24.52] | 1.00 | 11476 |
| Offset (*session* = 2 deviation) |  |  |  |  |
| Reward Learning Rate* | 1.17 | [-0.39, 2.69] | 1.00 | 14292 |
| Nonreward Learning Rate* | 0.30 | [-1.08, 1.69] | 1.00 | 13358 |
| Choice Consistency* | -0.49 | [-1.89, 0.88] | 1.00 | 11454 |
| *Bayesian Precision Weighting* | | | | |
| Base |  |  |  |  |
| Weight on Bayesian Prior^§^ | .61 | [.13, .95] | 1.00 | 28409 |
| Choice Consistency^†^ | 13.04 | [1.80, 26.73] | 1.00 | 27161 |
| Offset (*session* = 2 deviation) |  |  |  |  |
| Weight on Bayesian Prior* | 0.27 | [-1.13, 1.67] | 1.00 | 28900 |
| Choice Consistency* | -0.17 | [-1.56, 1.22] | 1.00 | 27363 |

*Note.* CI = credible intervals. ESS = effective sample size. ^§^Parameter values bound [0, 1]. ^†^Parameter values bound [0, ∞). *Parameter values on the untransformed, real scale.

For offset parameters, effects of repeated testing (*session* = 2) are considered credible if the 95% CI excludes 0. Here, none of the offset parameters across all three models indicated credible effects of repeated testing.

# Subjective Sleepiness: Linear mixed model estimates

Table S3

*Estimates of linear mixed model predicting log-transformed KSS scores*

| Predictor | B(SE) | 95% CI | p |
| --- | --- | --- | --- |
| Constant | .86 (.09) | [.69, 1.03] | <.001 |
| Condition | .63 (.11) | [.42, .84] | <.001 |
|  |  |  |  |
| No. observations | 68 | | |
| Marginal R^2^ | .28 | | |
| Conditional R^2^ | .46 | | |

*Note.* SE = standard error of B. CI = confidence intervals.

Significant predictors at *p<*.05 are **bolded**.

# Trial Accuracy: Linear mixed model estimates

Table S4

*Estimates of random-intercept logistic mixed model predicting trial accuracy*

| Predictor | B(SE) | 95% CI | Odds ratio | p |
| --- | --- | --- | --- | --- |
| Constant | .78 (.05) | [.69, .89] | 2.21 | <.001 |
| Condition | .01 (.04) | [-.08, .10] | 1.01 | .793 |
| Session | -.002 (.04) | [-.09, .08] | 1.00 | .956 |
|  |  |  |  |  |
| No. observations | 10050 | | | |
| Marginal R^2^ | *<.*001 | | | |
| Conditional R^2^ | .02 | | | |

*Note.* SE = standard error of B. CI = confidence intervals.

# Computational Modelling

We used weakly regularising priors for all group-level parameters to constrain the parameter space while allowing the data to drive posterior estimates. Specifically, we specified $\mathcal{N}(0, 1)$ for group-level means, and $Exp(1)$ for group-level standard deviations, with exceptions to solve issues with parameter estimate convergence and divergent transitions. In the Asymmetric Rescorla-Wagner model, all group-level SDs were specified with $Exp(.5)$, and for the Bayesian Precision Weighting model, group-level SDs for weight on prior ($\omega_{prior}$) and inverse temperature ($\beta$) were specified with $Exp(.1)$.

Table S3 and S4 provides the parameter estimate summaries for the standard Rescorla-Wagner and Bayesian Precision Weighting models, respectively.

Table S5

*Median and 95% highest density interval of Rescorla-Wagner model estimates*

| Parameters | Median | 95% Bayesian HDI | Convergence statistics | |
| --- | --- | --- | --- | --- |
|  |  |  | $\hat{R}$ | ESS |
| Base |  |  |  |  |
| Learning rate ($\eta$) | 0.84 | [.76, .90] | 1.00 | 5351 |
| Choice Stochasticity ($\beta)$ | 6.18 | [5.09, 752] | 1.00 | 6252 |
| Offsets |  |  |  |  |
| Learning Rate ($\Delta\eta$) | 0.20 | [-.12, .61] | 1.00 | 3064 |
| Choice Stochasticity ($\Delta\beta$) | -.10 | [-.22, .02] | 1.00 | 4900 |

*Note.* CI = credible interval. ESS = effective sample size.

Offset parameter values are reported on the untransformed real scale, and SR effects are deemed credible if 95% Bayesian CIs for these parameter estimates exclude 0. Weight on Bayesian *likelihoods* ($\omega_{evid})$ are transformed from $\omega_{prior}$, and hence not reported here.

Table S6

*Median and 95% highest density interval of Bayesian Precision Weighting model estimates*

| Parameters | Median | 95% Bayesian CI | Convergence statistics | |
| --- | --- | --- | --- | --- |
|  |  |  | $\hat{R}$ | ESS |
| Base |  |  |  |  |
| Weight on Bayesian *prior* ($\omega_{prior})$ | .62 | [.56, .68] | 1.00 | 4438 |
| Choice Stochasticity ($\beta)$ | 12.42 | [10.35, 14.60] | 1.00 | 6023 |
| Offsets |  |  |  |  |
| Weight on Bayesian *prior* (${\Delta\omega}_{prior})$ | -.05 | [-.20, .11] | 1.00 | 6943 |
| Choice Stochasticity ($\Delta\beta$) | -.10 | [-.25, .03] | 1.00 | 14888 |

*Note.* CI = credible interval. ESS = effective sample size.

Offset parameter values are reported on the untransformed real scale, and SR effects are deemed credible if 95% Bayesian CIs for these parameter estimates exclude 0. Weight on Bayesian *likelihoods* ($\omega_{evid})$ are transformed from $\omega_{prior}$, and hence not reported here.

## Posterior Predictive Checks

Figures S1A-C provides the average learning curves approximated by each model, as a function of *n* trials since the last reversal on the PRLT, separated by *condition*. In each sub-figure, shaded bands denote 95% confidence intervals. Note that although the Bayesian Precision Weighting model learning curve showed stronger visual alignment with aggregated participant accuracy, formal model comparison using WAIC, a metric of out-of-sample prediction accuracy, favoured the Asymmetric Rescorla Wagner model. This suggests the Asymmetric Rescorla Wagner model provided a better balance between fit and generalisability, even if its group-level trajectory slightly diverged from the observed mean in the current data. Visual agreement between model and participant curves can be informative, but it does not always reflect model adequacy in making forward predictions.

Figure S1

(A) *Rescorla-Wagner model*


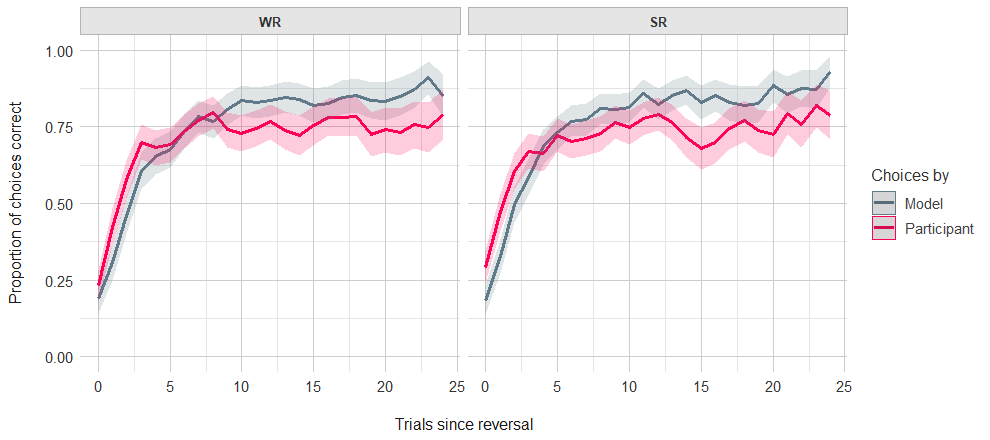


(B) *Asymmetric Rescorla-Wagner model*


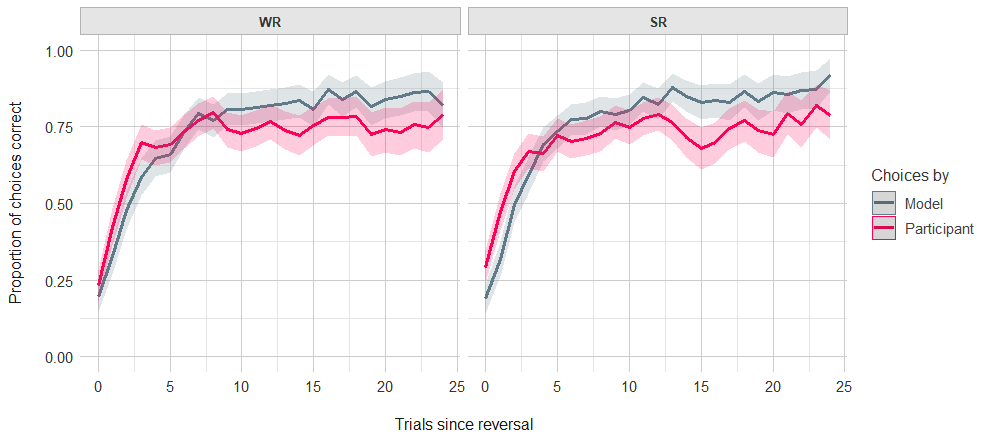


(C) *Bayesian Precision Weighting model*


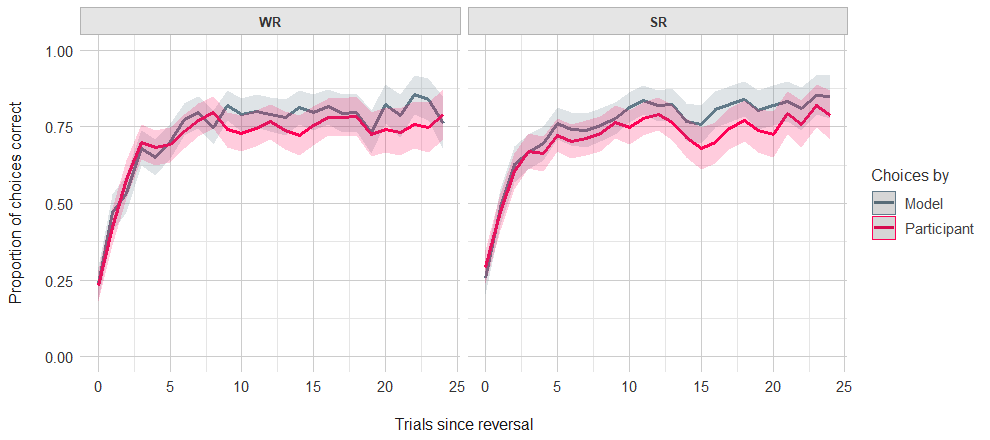


Figures S2A-C provides the proportion of responses matched by each model for each participant, separated by *condition.* Error bars denote 95% confidence intervals. Vertical dashed lines indicate chance-level predictions at 50%.

Figure S2

(A) *Rescorla Wagner model*


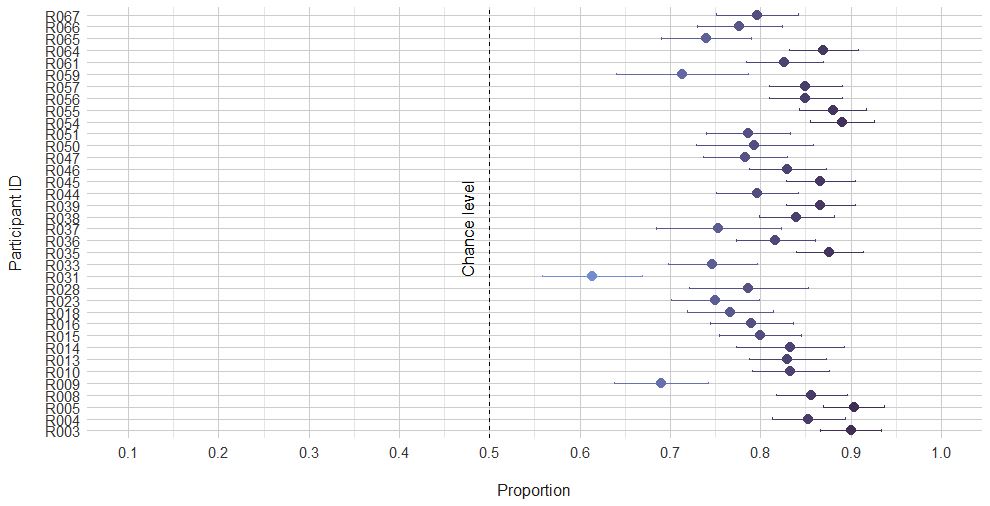


(B) *Asymmetric Rescorla Wagner model*


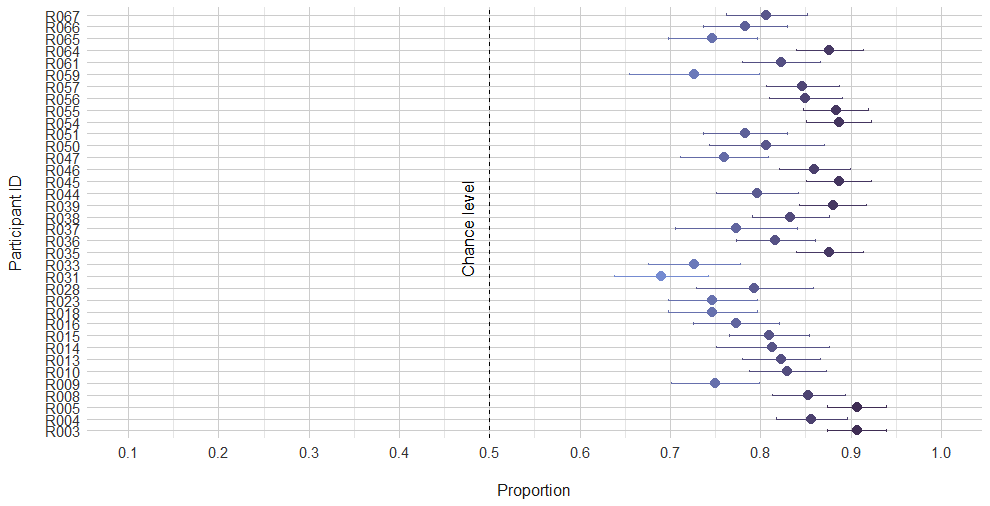


(C) *Bayesian Precision Weighting model*
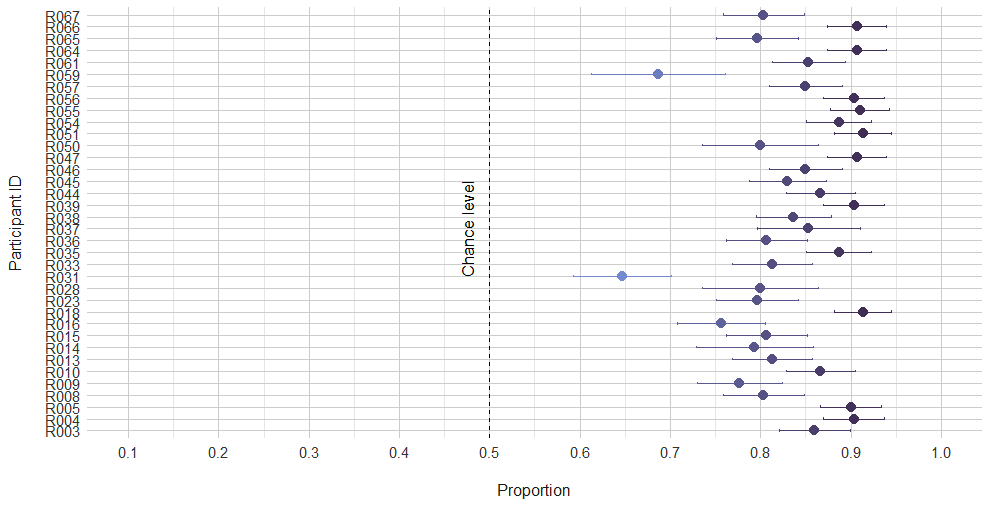

Supplement: SEQ_suppmat_R1_zsaf354 [file seq_suppmat_r1_zsaf354.docx]
